# A novel method for skin marking in radiotherapy: first clinical use of temporary organic tattoo seal

**DOI:** 10.1093/jrr/rrab126

**Published:** 2022-01-21

**Authors:** Masaaki Goto, Yoshiko Oshiro, Yoshio Tamaki, Toshiki Ishida, Yuichi Kato, Kazuya Shinoda, Hideyuki Sakurai

**Affiliations:** Department of Radiation Oncology, University of Tsukuba, 2-1-1, Amakubo, Tsukuba, Ibaraki, 305-8576, Japan; Department of Radiation Oncology, University of Tsukuba, 2-1-1, Amakubo, Tsukuba, Ibaraki, 305-8576, Japan; Tsukuba Medical Center, 1-3-1, Amakubo, Tsukuba, Ibaraki, 305-8558, Japan; Department of Radiation Oncology, University of Tsukuba, 2-1-1, Amakubo, Tsukuba, Ibaraki, 305-8576, Japan; Ibaraki Prefectural Central Hospital, Ibaraki Cancer Center, 6528, Koibuchi, Kasama, Ibaraki, 309-1793, Japan; Department of Radiation Oncology, University of Tsukuba, 2-1-1, Amakubo, Tsukuba, Ibaraki, 305-8576, Japan; Ibaraki Prefectural Central Hospital, Ibaraki Cancer Center, 6528, Koibuchi, Kasama, Ibaraki, 309-1793, Japan; Tsukuba Medical Center, 1-3-1, Amakubo, Tsukuba, Ibaraki, 305-8558, Japan; Ibaraki Prefectural Central Hospital, Ibaraki Cancer Center, 6528, Koibuchi, Kasama, Ibaraki, 309-1793, Japan; Department of Radiation Oncology, University of Tsukuba, 2-1-1, Amakubo, Tsukuba, Ibaraki, 305-8576, Japan

**Keywords:** External radiotherapy, skin marking, temporally tattoo, quality assurance

## Abstract

An oil-based pen is widely used as a skin marker for identification of the isocenter and computed tomography (CT)-coordinate origin during radiotherapy. However, use of this pen has some disadvantages, including color loss and color migration. To address these problems, we have developed use of a temporary fashion tattoo (Inkbox) for skin marking. The utility and feasibility of Inkbox as an alternative to an oil-based pen were evaluated in this study. The study included patients from two centers who required skin marking for radiotherapy performed between December 2020 and March 2021. Skin markings were made with an oil-based pen or with Inkbox. The durability was recorded during daily irradiation. Skin markings with Inkbox were made in 32 patients. The total number of skin markings was 94: 64 with Inkbox and 30 with an oil-based pen. A questionnaire survey to evaluate each method was conducted among patients after radiotherapy. The median durations of marking were 16 and 4 days with Inkbox and an oil-based pen, respectively (p-value < 0.001). The survey showed that Inkbox had less impact on the daily lives of patients, including reduced color migration to clothes and less concern about disappearance of the marking. There were no adverse cutaneous side effects with Inkbox. The duration of marking with Inkbox is about 16 days, with little impact on daily life. These findings suggest that Inkbox is a potentially useful method of skin marking in radiotherapy.

## INTRODUCTION

Skin marking is an important process for ensuring the reproducibility of the radiotherapy setup [1]. Radiological technologists usually draw lines on the body surface at the time of treatment planning computed tomography (CT) and the first irradiation for positioning. Positional information, such as the coordinate origin, isocenter and irradiation field, marked on the body surface enables accurate treatment delivery through the course of radiotherapy.

There are several methods available for skin marking and each has advantages and disadvantages [2]. Marking with oil-based pens is widely accepted in Japan as an easy, non-invasive and cost-effective method, but the mark is very easy to rub off and fades after only a few days. Thus, patients have to take care of the skin marks and have bathing restrictions and color migration to clothes. A faded skin mark is also a problem for radiological technologists because patient positioning and redrawing of lines becomes more time consuming. This problem is worsened if there is a long period from treatment planning to the start of treatment, which often occurs in Japan due to holidays and vacations, and this makes it difficult to manage skin markings. In this situation, transferable stickers such as Field Marker® (Chubu medical co., Japan) that last longer than oil-based pens are preferably used [3]. These stickers have the advantage of being noninvasive. A thin film of the mark is wetted with water and transferred to the body surface; however, this mark may be easily peeled off. Therefore, patients need to be careful not to rub the mark. Permanent tattoos using needles and ink and temporary henna tattoos can also be used, but these approaches are limited by problems of invasiveness for patients and additional work for radiological technologists.

Inkbox® (Inkbox, Canada) is a temporary organic tattoo that was developed as a fashion accessory. By coloring directly on the collagen of the skin, the mark on the seal is expected to last for 10–18 days [4,5]. This mechanism gives resistance to fading from friction and perspiration, and thus, Inkbox may function for skin marking in radiotherapy. We collaborated with Inkbox Japan to develop a prototype of an Inkbox product specifically for this purpose. This study is the first to investigate Inkbox for skin marking during radiotherapy.

**Fig. 1.**
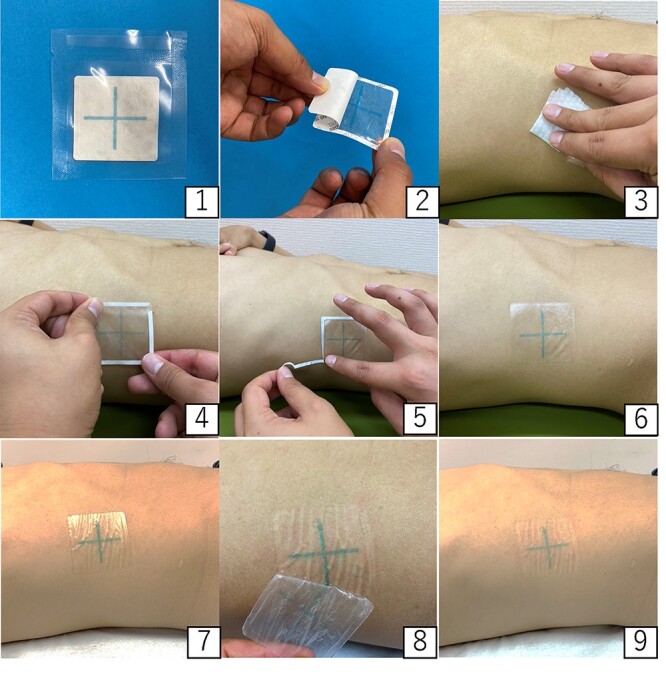
An exemplary method of applying skin marking using Inkbox. 1. Package of Inkbox. 2. Peel off the center bottom layer. 3. Wipe skin. 4. Attach Inkbox on the site. 5. Peel off the surrounding bottom layer. 6. Inkbox is successfully applied. 7. Wait an hour. 8. Remove Inkbox from the skin. 9. The mark is transferred to the skin.

**Fig. 2.**
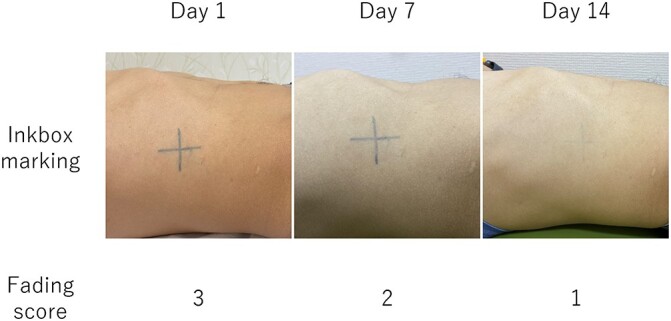
An example of the change in the Inkbox mark. On Day 1, the color of the mark got darker and the fading score was 3: visible. On Day 7, the fading score was 2: faded but visible. On Day 14, the fading score was 1: faintly visible.

Photographs of the process of skin marking using Inkbox are shown in Fig. 1. The adhesive seal has a pattern made with the ink of Genipa, which directly colors collagen in the skin. After adherence of this seal to the skin for one hour, the pattern appears on the skin. The color of this ink is light blue and the initial appearance of the transferred pattern is also light blue, but the color darkens in a few days and becomes deep blue. An example of the change in the Inkbox mark with the author as the subject is shown in Fig. 2. Once the skin is colored, the mark is expected to last longer than a mark made with an oil-based pen.

## MATERIALS AND METHODS

Patients from two hospitals who were scheduled for radiation therapy and skin marking between December 2020 and March 2021 were included in the study. Patients with a history of contact dermatitis to adhesive dressing materials, contact dermatitis to alcohol, allergic reaction to nuts or berries and those who are considered to have difficulty in understanding the purpose of this study were excluded. The study was approved by the institutional review boards at the two hospitals and written informed consent was obtained from all participants.

Inkbox was used for some or all skin markings that referred to the origin of treatment planning CT, as an alternative to an oil pen. Confirming Inkbox mark after an hour by a radiological technologist is desirable, but not mandatory, which depends on patients and situations. Most of the patients removed the seal by themselves. After the start of radiotherapy, skin marking that referred to the isocenter was lined by oil-based pen as usual because Inkbox is difficult to retouch and the safety of a second application was unknown. After the start of treatment, the radiological technologist observed the markings daily and recorded a fading score of 3: visible, 2: faded but visible, 1: faintly visible, or 0: invisible. If marks had faded and received a score of 1, the radiological technologist reapplied the mark with an oil-based pen. These events were also recorded. A questionnaire survey on skin marking was given to the patients during or after completion of radiotherapy.

This is the first use of Inkbox in radiotherapy and we worked with Inkbox Japan to develop the product for radiotherapy. Inkbox was originally designed for making temporary tattoos for fashion and is not classified as a pharmaceutical or medical product. Before the study, the optimal size and pattern were investigated and 3 sizes and patterns were used in the study: a square of 5 × 5 cm with a cross pattern, a rectangle of 5 × 12 cm with a line pattern and a rectangle of 5 × 12 cm with a cross pattern. The width of the line was 2 mm.

The duration of skin marking was defined as the time from initial marking to that when the fading score was 1. The results for Inkbox and the oil-based pen were compared by Man-Whitney U-test. Univariate analysis of the durability of Inkbox marking was performed for age, gender, mark site and institution. All statistical analyses were performed with EZR, which is a graphical user interface for R [6]. To investigate patient satisfaction, a questionnaire was used after radiotherapy. The patients evaluated the marking method, impact on daily life and duration of marking with Inkbox and an oil-based pen using four levels from 1 = unsatisfactory to 4 = satisfactory.

## RESULTS AND DISCUSSION

**Fig. 3.**
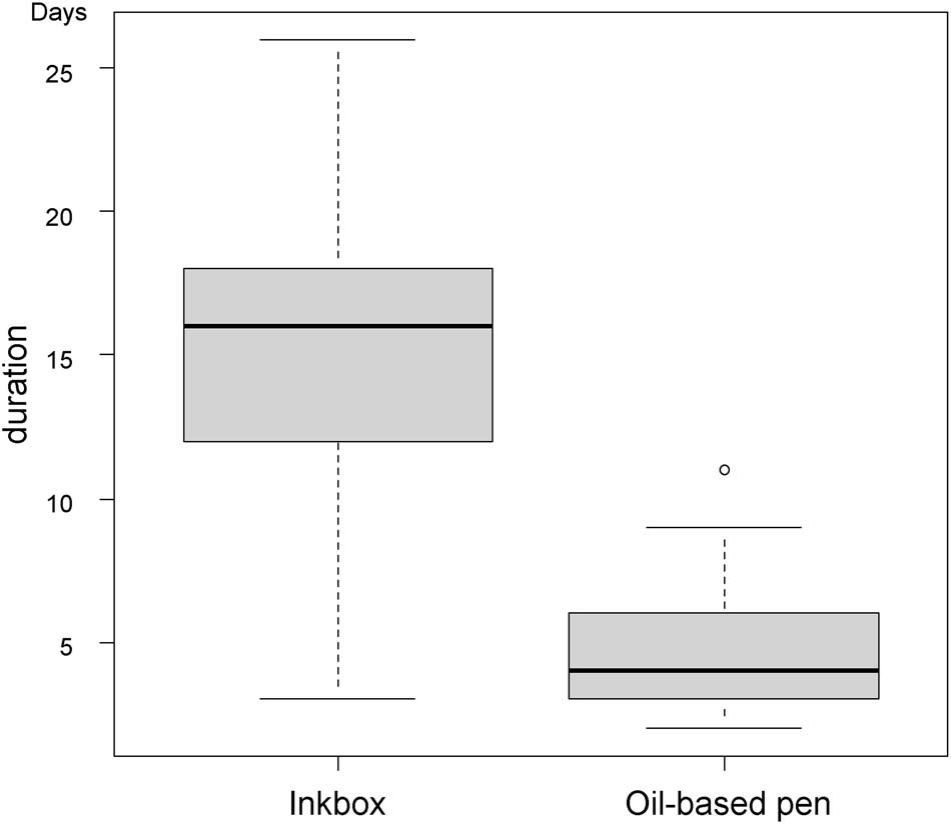
Result for the duration of Inkbox and oil-based pen compared by Man-Whitney U-test. The median duration of skin marking (median: 4; Q1: 12 – Q3: 18 days) was significantly longer than that of oil-based pen (median: 4; Q1: 3 – Q3: 5.8 days) (U = 1860, p-value < 0.001).

A total of 32 patients were enrolled in the study at the two centers between December 2020 and March 2021. A total of 94 sites were marked: 64 with Inkbox and 30 with an oil-based pen. The baseline characteristics are shown in Table 1. The result for marking duration is given in Fig. 3 and univariate analysis for Inkbox is given in supplementary data. The median duration of skin marking was significantly longer with Inkbox (median: 16; Q1: 12 – Q3: 18 days) compared to use of an oil pen (median: 4; Q1: 3 – Q3: 5.8 days) (U = 1860, p-value <0.001). In univariate analysis, age < 60 years was significantly related to a prolonged duration (p = 0.0137), but gender, mark site and institution were not significant.

**Table 1:** The baseline characteristics

All patients		32
Institutions	Ibaraki Prefecture Central Hospital (IPCH)	15
	Tsukuba Medical Center (TMC)	17
Sex	Female	14
	Male	18
Age		67.4(38–85)
All marking sites		94
Types of marking	Inkbox	64
	Oil-base pen	30
Site of marking	Pelvic	38
	Chest-abdominal	55

The results of the questionnaire indicated that patients tended to view Inkbox as more satisfactory than an oil-based pen in terms of marking method and impact on life during treatment. The average scores (on a 4-point scale) for satisfaction with the marking method, impact on daily life and duration were 3.8 vs 3.6, 4.0 vs 3.8 and 3.5 vs 3.7 for Inkbox vs an oil-based pen. Thus, all patients indicated ‘4 (satisfactory)’ for Inkbox with regard to impact on daily life. There were no adverse events related to skin markings that interrupted radiotherapy. There were a few cases of mild skin redness after the Inkbox seal was removed, but no additional treatment was needed.

The main advantage of Inkbox is its duration of marking. To our knowledge, 16 days is the one of the longest duration recorded among currently reported noninvasive skin marking methods. This period is almost the same as the turnover period of the stratum corneum, which is consistent with the mechanism of Inkbox [7]. The longer duration is useful in cases in which longer periods are required from planning CT to the start of radiotherapy, and is also beneficial for radiological technologists because less reapplication of skin markings is needed. The second advantage is that patients do not have to worry about the mark. The color is directly applied to the stratum corneum, which eliminates concerns of color transfer to clothing or mark fading. This is particularly beneficial in hot and humid climates, in which people tend to sweat and frequently take a shower or bath. However, it is difficult to mark certain areas using Inkbox, including a hairy area, such as the front mark for prostate irradiation, and a curved surface, such as that marked for breast irradiation and additional care is required for Inkbox markings in these regions.

A henna tattoo also has a long duration and is used as a method of staining the superficial skin layers in some countries. The duration of a henna tattoo is reported to be 23 days [8], which is longer than that with Inkbox. However, a henna tattoo needs a long drying period (12–30 minutes) to fix the color [2], which increases the workload on a radiological technologist. Thus, using Inkbox is easier than creating a henna tattoo and less burdensome for the radiological technologist. Transferable markers are also used in Japan. These are easily scratched off, but last longer than oil-based pens. We did not compare Inkbox with transferable markers, and further investigations of the best use of each marker are warranted. Technologies such as image-guided radiation therapy (IGRT) using cone-beam computed tomography (CBCT) and 3D surface imaging have also recently emerged as methods for positioning, and skin marking may not always be necessary [9,10]. However, not all facilities use these techniques and the importance of skin marking is still high. The development of a more durable and convenient skin mark is warranted in the radiotherapy field.

The patients rated Inkbox better in terms of marking method and impact on their lives. In particular, the impact on daily life received the highest rating in all cases, suggesting that Inkbox has little impact on daily lives. The absence of restrictions on bathing or showering and the lack of color transfer to clothes are likely to explain these positive results. For duration, the rating by the patients was contradictory to the actual findings for duration. This may be because some Inkbox markings were barely visible if Inkbox stickers were not well fixed. These marking failures may have led to the less positive evaluations by patients.

The limitations of the study include the small number of facilities and patients, and the non-randomized method of selecting the marking site. It is also important to note that Inkbox was not developed for medical purposes. Although there has been reported contact dermatitis caused by the component of Inkbox [11], we observed no adverse effects related to Inkbox skin marking in the study. The Inkbox sticker is somewhat stiff and sticky, and development of a softer, more skin-friendly base material is needed. The patterns and sizes of the stickers were also limited, and establishment of a variety of patterns and lines to meet users’ requests is warranted. Within these limitations, we conclude that Inkbox provides a novel skin marking method in radiotherapy. The duration of Inkbox marking is about 16 days, with little impact on the daily life of the patient. Thus, Inkbox has considerable potential as a method of skin marking in radiotherapy.

### ACNOWLEDGEMENTS

This work was supported by University of Tsukuba.

## CONFLICT OF INTEREST

Inkbox products were provided free of charge from Inkbox Japan.

## PRESENTATION AT A CONFERENCE

This study was presented at the 34th Annual Meeting of the Japanese Society for Radiation Oncology.

## Supplementary Material

Supplementary_data_rrab126
