# Supplementary material for: A novel method for skin marking in radiotherapy: first clinical use of temporary organic tattoo seal
Source: J Radiat Res. 2022 Jan 21;63(2):314–8. doi: 10.1093/jrr/rrab126 (PMC8944313; doi:10.1093/jrr/rrab126)
Supplement: Supplementary_data_rrab126 [file supplementary_data_rrab126.docx]

Supplementary data

Univariate analysis of Inkbox duration

|  |  | Mean duration | P-value |
| --- | --- | --- | --- |
| Age | <60 | 17.2 ± 3.02 | 0.0137^¶^ |
|  | ≥60 | 14.2 ± 4.82 |  |
| Sex | Female | 16.4 ± 4.06 | 0.0784 |
|  | Male | 14.4 ± 4.68 |  |
| Institutions | IPCH | 15.9 ± 4.13 | 0.452 |
|  | TMC | 14.9 ± 4.66 |  |
| Site of marking | Pelvic | 14.5 ± 4.62 | 0.22 |
|  | Chest-abdominal | 15.9 ± 4.38 |  |
